# Pellagra, a re-emerging disease: a case report of a girl from a community ravaged by insurgency

**DOI:** 10.11604/pamj.2019.33.195.17494

**Published:** 2019-07-12

**Authors:** Ahmadu Baba Usman, Pembi Emmanuel, Dogo Belmont Manchan, Akoma chinyere, Ovansa Emmanuel Onimisi, Mava Yakubu, Kenji Hirayama

**Affiliations:** 1 Department of Paediatrics, Federal Medical Centre Yola, Adamawa State, Nigeria; 2 Program for Nurturing Global leaders in Tropical and Emerging Communicable Diseases, Graduate School of Biomedical Sciences, Nagasaki University, Japan; 3 Adamawa State Ministry of Health, Nigeria; 4 Department of Immunogenetics, Institute of Tropical Medicine (NEKKEN), Nagasaki University, Nagasaki, Japan; 5 National Youth Service Corps, Abuja, Nigeria; 6 Department of Pediatrics, Bingham university Abuja, Nigeria

**Keywords:** Pellagra, children, nutrition, dermatitis, diarrhea, conflict

## Abstract

Pellagra is a nutritional disorder of niacin deficiency which is characterized by triad of dermatitis, diarrhea and dementia. It is often seen in a state of poor nutrition among alcoholics, homeless and patients suffering from malabsorption. Though seldom occurs in children, its re-emerging is seen as a result of worsening food security in vulnerable population during conflict or insurgency. We report the case of 12-year-old female pastoralist who presented darkening and thickening of the hands, feet, ankles, neck and her upper trunk. Conflicts and insurgency usually occur in resource constraint settings where health workers are few and overworked. Therefore, continuously educating health workers and the general public regarding nutrition and its disorders like pellagra is a priority. Public Health authorities and policy makers also ought to take pediatric nutrition serious in order to avoid its escalation in internally displaced persons or children orphaned by insurgency.

## Introduction

A Spanish physician Gaspar Casal first described Pellagra in 1735 [1]. It is a nutritional disease which often results from deficiency of niacin which manifests with classic triad of dermatitis, diarrhea and dementia [2]. It is commonly associated with malnutrition and extreme poverty [3]. Though many were killed by it in the United States in the past, it has now been eradicated due to remarkable advancement in public health and nutrition through enrichment of wheat flour with nicotinic acid [4] and thus only isolated cases among alcoholics, drug abusers, food faddists and homeless people are seen [5]. It often occurs in middle-aged but seldom below the age of 15 y, and never in infants and young children [6]. We present a rare case of pellagra in a pastoralist girl of a low socio-economic background from Adamawa State, North-eastern Nigeria. This report unveils the reemergence of pellagra as consequence of worsening food insecurity from persistent conflicts and insurgency associated with Boko-Haram in North-Eastern Nigeria. It also seeks to inform public health authorities and policy makers of the urgent need to address nutrition issues in the camps of persons internally displaced by insurgent to avoid the escalation of disease.

## Patient and observation

She is a 12-year-old female pastoralist who presented to the federal medical Yola with 7 weeks history of well-defined darkening and thickening of the hands, feet, ankles, neck, and the upper trunk region including upper chest and back. She had similar lesions but milder form on lips and periorbital region. The affected area of the skin was said to get exacerbated after exposure or re-exposure to sunlight. The lesions progressively increased in size since 5 weeks with accompanied itching burning sensations following exposure to the sun. She also complaint of recurrent episodes of passage of loose watery stools. As a pastoralist on perpetual travel without school enrolment, it was difficult to access her academic performance. Questions to enquire about changes in her intelligence or cognitive abilities did not reveal remarkable derangement. There are, however, features of dementia like subtle memory change, limited social and thinking abilities, nervousness and apathy. She had sought for medical intervention several times in many peripheral clinics but no significant improvement was noticed. She had no history severe anger, rage, delusions or convulsions. There was no any history of tuberculosis treatment. Her dietary history revealed a persistent intake of corn which the basic meal in the household. Patient was worried and less active as the disease condition worsened.

**Figure 1:**
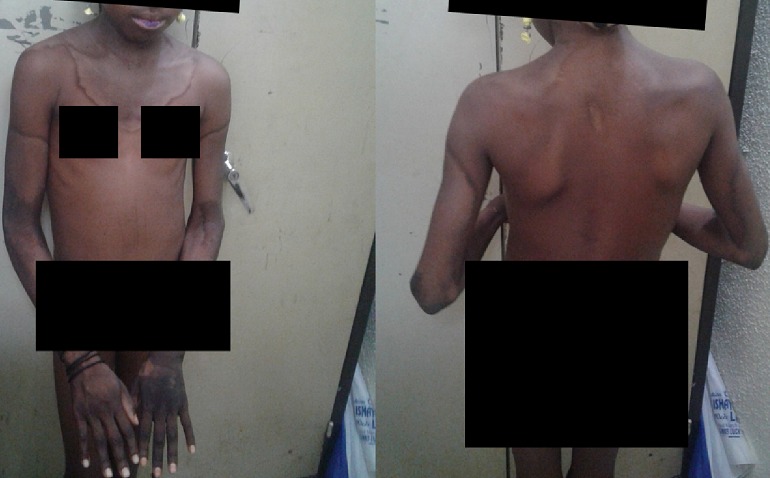
Typical case of the patient with pellagra seen at the federal Medical Centre Yola, Nigeria

On examination, a depressed young girl with a well demarcated symmetrical hyper pigmented and thickened burn-like eruptions with erythematous margins covering the neck region and extending on to the upper part of the trunk including the thorax and back are seen. These herperkeratinised and thickened hyperpigmentation lesions also appeared symmetrically over the dorsum of the feet, the ankles, the upper part of the legs, the hands, wrist, arms forearms and milder forms on the lips (Figure 1). She also had koilonychias. Her anthropometric weight and height revealed that she was wasted and stunted for age with less than -2 but greater than -3 WHO Z- score. Based on the history and clinical findings of photosensitive casal necklace and acral dermatitis, a diagnosis of Pellagra was made. Patient could not afford to conduct requested laboratory investigations due to the family social standing. She was then placed on nicotinamide 100 mg with multivitamin supplements. Patient improved rapidly after commencement of medication thus supporting our clinical diagnosis. Dietary advice to consume food rich in niacin which the family can afford such as legumes, fish, vegetables etc were elaborated However, complete follow up of the patient to see her state of full recovery became impossible as the patient left the hospital and did not show for follow up.

## Discussion

Pellagra is a disease which results from a deficiency of niacin or vitamin B3. These may arise from poor dietary intake as occurs in poverty-stricken areas where only maize (corn) is consumed [7]. It may as well be as a result of deficiency in tryptophan, a substance prevalent in legumes, fish and meats [2]. Other causes may be due to inadequate and/or decreased absorption of the vitamins containing food or ineffective processing of the food containing the vitamin. Our patient is from a low socio economic background whose food has constantly been corn which is the cheapest that the people with low income can afford since it is commonly produced by subsistence farmers. Her nutrition is also poor in terms of meal frequency 2-3 times/day, no nutritious snacks in between meals and her meal diversity is extremely narrow, majorly corn and fresh boiled cow milk. This is far less than the acceptable meal diversity. In addition, poor meal preparation of meal by applying so much heat can damage the limited micro nutrients available. Her anthropometric weight and height for age which were less than -2 but greater than -3 WHO Z- score signifies the girl was wasted and stunted which are evidence of existence of both macronutrient and micro nutrient deficiency. Similarly, the Koilonychias further confirms her micro nutrient deficiency because iron is also a micro nutrient and its deficiency leads to development of spoon shaped nails. Pellagra also occurs rarely as complication from isoniazid therapy [8]. Other situations which may deplete niacin leading to its deficiency is increased tryptophan metabolism as in HIV, in which it stimulates a pellagra-like condition where tryptophan levels is decreased [9]. Carcinoid syndrome also causes a pellagra-like disorder [10].

Though necessary investigation to confirm this diagnosis had not been carried out, however, through meticulous history and physical examination, a diagnosis was made. Thus our diagnosis was mainly clinical from observed recovery following niacin treatment. The skin changes that started occurring were major pointers to this disease. The skin lesions of the patient were characteristic of pellagra. The associated diarrhea and mild change in mental state (depression, worry) explained the classic triad of dermatitis, diarrhea and dementia. Her condition was diagnosed only clinically due to financial constraints. Niacin assay is very expensive and not a routine investigation in the hospital. In most cases, this assay is carried out for research purpose. In this resource poor environment, this test is almost if not impossible to run. However, other findings in routine investigation such as anemia are a pointer to chronic malnutrition. Though drug eruptions may mimic pellagra, the history debunked photosensitivity reactions since there was no history of medication prior to presentation. Another close differential, Hartnup disease, a condition in which tryptophan is not absorbed effectively can be confirmed with tools for genetic analysis. Even though such tools were lacking, this patient's condition is not likely to be it since Hartnub disease presents much earlier in life. Additionally, there was no any family history of similar condition. Since Hartnup disease is autosomal recessive disorder, there is likelihood of finding such history if she has the disease. Other conditons that may resemble pellagra includes systemic lupus erythematosus (SLE) flares which often affect the malar region rather than the neck and acral regions as in this patient. Additionally, this patient hadn't signs to fulfill diagnostic criteria for SLE.

## Conclusion

Pellagra is re-emerging as a result of worsening food insecurity due to conflict or insurgency. The vulnerable population tends to be children who cannot fend for themselves in situation where they are orphaned by insurgency. There is an urgent need for public health authorities and policy makers to take nutrition issues seriously especially in cases of internally displaced persons and orphans. Health workers in rural settings should be equipped with knowledge to be able to diagnose some disease such as pellagra amidst lack of diagnostic facilities.

## Competing interests

The authors declare no competing interests.

## References

[1] Hui S, Heng L, Shaodong W, Fangyu W, Zhenkai W (2017). Pellagra affecting a patient with Crohn's disease. An Bras Dermatol.

[2] Meyer-Ficca M, Kirkland JB (2016). Niacin. Adv Nutr.

[3] Li R, Yu K, Wang Q, Wang L, Mao J, Qian J (2016). Pellagra Secondary to Medication and Alcoholism: a case report and review of the literature. Nutr Clin Pract.

[4] Karthikeyan K, Thappa DM (2002). Pellagra and skin. Int J Dermatol.

[5] Galimberti F, Mesinkovska NA (2016). Skin findings associated with nutritional deficiencies. Cleve Clin J Med.

[6] Naveen KN, Pai VV, Bagalkot P, Kulkarni V, Rashme P, Athanikar SB (2013). Pellagra in a child--a rare entity. Nutrition.

[7] Villaverde Doménech ME, Simón Sanz E, Pujol Marco C, Pérez del Caz MD, Blanco Cerdá O, Safont Albert J (2014). Pellagra: a challenging differential diagnosis in burn injuries. J Tissue Viability.

[8] Post FA (2016). Pellagra: a rare complication of isoniazid therapy. Int J Tuberc Lung Dis.

[9] Tremeschin MH, Cervi MC, Camelo Júnior JS, Negrini BV, Martinez FE (2007). Niacin nutritional status in HIV type 1-positive children: preliminary data. J Pediatr Gastroenterol Nutr.

[10] Bell HK, Poston GJ, Vora J, Wilson NJ (2005). Cutaneous manifestations of the malignant carcinoid syndrome. Br J Dermatol.

